# Would Biopsy Change the Management of Kidney Cancer in the Coming Decade? The Role of Biopsy in Small Renal Masses: A Narrative Review

**DOI:** 10.3390/cancers17244032

**Published:** 2025-12-18

**Authors:** Łukasz Dariusz Lisak, Paweł Hełka, Aleksandra Kaźmierczak, Anna Kaletka, Sarah Sampers, Piotr Kania, Miłosz Jasiński, Maciej Salagierski

**Affiliations:** 1 Urology Department, Collegium Medicum, University of Zielona Góra, 65-417 Zielona Góra, Poland; 2 Urology Department, Mazovian Hospital, 08-110 Siedlce, Poland

**Keywords:** kidney cancer, renal cell carcinoma, renal mass biopsy, renal tumor biopsy

## Abstract

Small renal masses (SRM, ≤4 cm) are detected more frequently owing to better imaging techniques, which in turn causes a problem of overdiagnosing and unnecessary surgical treatment, including avoidable excision of benign lesions, resulting in nephron loss and decline in renal function. Renal mass biopsy (RMB), either a core needle biopsy (CNB) or fine needle aspiration biopsy (FNAB), is a safe procedure with a general complication rate usually <10%, clinically negligible risk of tumor spreading (0.01%), and high diagnostic efficiency (sensitivity of 95–99%, specificity up to 100%). RMB allows not only the distinction between benign and malignant lesions but also the evaluation of the histological subtype and tumor grading, which improves risk stratification and helps in choosing the proper treatment (including indications for active surveillance, nephron-sparing surgery, or ablation). Additional tests (IHC, molecular tests) are playing a more important role, considering the newest WHO 2022 classification, which considers molecularly defined tumors. Major scientific associations (AUA, EAU, NCNN) recommend considering RMB when the result may change patient management. Despite the solid data, RMB is still underused due to habits and a lack of standardized protocols. Wider implementation of RMB may limit excessive surgeries and improve the precision of management decisions.

## Simple Summary

A significant proportion of small renal masses are benign or low-grade malignant tumors. Renal mass biopsy (RMB) offers high diagnostic accuracy, reduces the need for unnecessary surgical management, and facilitates tailored treatment strategies by distinguishing benign from malignant lesions and identifying metastatic disease. Despite the potential benefits, it remains underutilized. The concerns about RMB are largely unfounded—it is a safe procedure with a low complication rate (<10%), and with advances in imaging and methods of pathological evaluation, its accuracy is acceptable. Broader implementation of RMB could significantly improve patient care by minimizing overtreatment and ensuring more precise therapeutic decision-making.

## 1. Introduction

Renal cell carcinoma (RCC) is the 14th-most common cancer worldwide, with 434,419 new cases recorded in 2022. It also ranks as the 16th-leading cause of death among cancers, with 155,702 deceased patients in 2022 [1]. Epidemiologic data indicate that the cumulative risk (%) among both sexes in the age group from 0 to 74 years is highest in more developed regions (1.5% incidence and 0.5% mortality for men and 0.7% incidence and 0.2% mortality for women), while the highest cumulative risk occurs in North America, then Western Europe and Central and Eastern Europe [2]. The growing incidence of kidney cancers may be partially attributed to an increased detection of these tumors, especially of presymptomatic small renal masses (≤4 cm), which can be attributed to advancements in radiological methods and a wider employment of abdominal imaging [3].

The traditional approach to suspicious renal masses relies largely on radiological imaging, on which diagnosis is based. To date, renal mass biopsy, such as core needle biopsy (CNB) and/or fine needle aspiration biopsy (FNAB), is not required before surgery. Renal mass biopsy (RMB) is currently advocated in the advanced tumor stages when radiological features suggest that complete resection is not possible. The biopsy is then performed prior to the initiation of chemotherapy. Biopsy is performed to confirm the diagnosis, classify subtypes, and guide oncological treatment [4,5]. Other indications for renal mass biopsy include the separation of indolent from aggressive tumors using molecular markers; concomitant with ablative therapies; exclusion of non-renal cell primary tumors (metastases and lymphoma); or benign conditions (such as abscess) that might not require surgery [6].

Despite advances in imaging and surgical techniques, the management of renal masses remains predominantly surgical (radical or partial nephrectomy). However, RCC mortality has remained relatively stable, indicating substantial overdiagnosis and overtreatment [5,7,8]. This is reflected by an 82% increase in surgeries for benign renal masses in the USA between 2000 and 2009 [7]. Such unnecessary procedures result in avoidable nephron loss, which is associated with renal insufficiency and reduced survival; therefore, surgical intervention should be reserved for patients in whom RCC is likely to affect life expectancy [5].

Considering the growing incidence of RCC and challenges it poses, the aim of our review is to discuss the evolving role of renal mass biopsy, its safety, diagnostic yield, current guidelines, and future perspectives.

## 2. Materials and Methods

A literature PubMED review was conducted with keywords renal cell carcinoma (RCC), renal mass biopsy (RMB), core needle biopsy (CNB), fine needle aspiration biopsy (FNAB), ROSE, guidelines, and perspectives.

## 3. Renal Mass Biopsy (RMB)–Safety Considerations

The history of renal needle biopsy begins in 1944 with a Swedish scientist and inventor, Nils Alwall [9,10]. He was successful in obtaining renal tissue by an aspiration technique from his first 13 patients. Unfortunately, 1 of them died from complications; therefore, he decided to no longer pursue the technique and did not publish his work until 1952 [10]. However, in 1951, Paul Iversen and Claus Brun from Copenhagen published their first series of renal biopsies, which prompted a number of physicians around the world to immediately attempt renal biopsy, using cutting as well as aspiration techniques [9,10].

Nevertheless, in terms of the management of solid renal masses, biopsy was not the default intervention. A traditional approach assumed surgical resection as the standard management of solid renal masses. This practice paralleled the rise in incidence of RCC, which unexpectedly corresponded to an increase in mortality. This prompted reexamination of the traditional approach, as the assumption that early intervention would lead to better survival was challenged [11]. The traditional approach was questioned, yet there were numerous concerns regarding the RMB procedure. These concerns include the safety of the procedure (possibility of tumor seeding along the biopsy tract) and diagnostic accuracy [3]. Isgrò et al. [12] found that out of 225 renal biopsies conducted for tumors, the seeding of the tumor was reported only once (the seeding rate of 0.4%) in a cT1a RCC, and the patient underwent robot-assisted partial nephrectomy. The pathology showed tumor seeding around the needle trajectory, confirming a T4-stage tumor. The follow-up was negative (29 months). Other research carried out also confirmed very low, if any, tumor seeding during the RBC: 0% (0/168) by Gao et al. [4], 0% (0/509) by Richard et al. [13], and 0% (0/94) by Bada et al. [14]. More importantly, a systematic review of 57 studies, 1946–2014 by Marconi et al. [15] indicated just one case of tumor seeding (1/5228, the seeding rate 0.0019%). The overall estimated risk of tumor seeding along the needle tract is <0.01% and the risk increases with the number of needle passes and with non-cutting needles [6]. Therefore, the risk of the tumor spreading, affecting the long-term survival of the patient, remains negligible. Other reported complications are perirenal and skin hematomas, venous bleeding through coaxial sheath, hemorrhage, hematuria, nausea/vomiting, dizziness, persistent pain, urinary retention, fever, prolonged inpatient stay, and infections [4,12,13].

An overall complication rate reported by Isgrò et al. [12] is 4.8%; by Serhal et al. [16] is 5%, with perinephric hematoma (3%, 6/167) being the most common complication; 8.5% by Richard et al. [13] (48 adverse events in 42 out of 492 patients with the post procedure complications status available), again with perirenal hematomas accounting for most of the postoperative adverse events (75% of adverse events, 4.7%, 24 cases); while Gao et al. [4] reported hemorrhage as the most common side effect (3%, 5/168).

The available data suggest that the RMB is a safe procedure, with an overall complication rate below 10% (with a study by Tong et al. [17] having an 18.1% rate), varying between studies conducted (Table 1).

## 4. Diagnostic Accuracy

According to current knowledge, renal biopsy can provide a definitive diagnosis in 95% of cases [12]. A recent paper by Mazin Hashim et al. [18] estimated the overall sensitivity to be 95% (95% confidence interval [CI] 90–98) and specificity of 100% (95% CI 95–100) in the case of RMB. The authors examined 193 patients with SRM who underwent RMB. The positive predictive value was 100%, and the negative predictive value was 92%. The concordance rate between the histology of the RMB and final histology was calculated to be 89%. Moreover, treatment was withheld in 67 patients due to the RMB results suggesting benign tumors or being inconclusive. Gao et al. [4] examined the success rate of SRM core needle biopsy and established that CNBs achieved a specific histologic diagnosis in 86.9% (146/168) of SRMs in their study. CNBs not only provided histological evidence of malignancy versus benignity but also provided prognostic information such as tumor subtypes and nuclear grading. The achieved accuracy of tumor typing was 100%, subtyping of 97.3–100% and nuclear grading for CCRCC of 83.8%. Marconi et al. [15] estimated an overall median diagnostic yield of 92% for the diagnosis of malignancy in their systematic review of 57 studies. In the Azawi et al. [21] study, 61 patients underwent surgical treatment. In 60 of these, the initial RTB diagnosis was confirmed. In one patient, the initial RMB was inconclusive, while a renal cell tumor was found in the pathological specimen. This resulted in a sensitivity of the core biopsies of 98% (56/57) and a specificity of 100% (4/4). This issue was also addressed by Tong et al. [17], who examined the role of FNAB in the management of SRMs. Out of 169 qualified patients, 83 received FNAB before surgery. The initial success rate of FNAB was 91.6% (76/83), and the rate of accuracy in identifying malignancies was 100%. Bada et al. [14] achieved similar results: the initial biopsy was diagnostic in 93.6% (*n* = 88) of patients. Of these, 67 (76.1%) were malignant and 21 (23.9%) were benign. Agreement between biopsy and surgical histology was found in 94% of cases. Jasinski et al. [22] performed 387 RMBs from January 2016 to June 2020. The mean size of the analyzed masses was 47.8 mm. In total, 56% of tumors ≤ 40 mm (247) and 8% of tumors > 40 mm (64) were biopsied. In addition, 76 RMBs of disseminated tumors were performed. Out of the biopsies performed, 14.9% of RMBs were non-diagnostic, and as many as 27.1% of RMBs of tumors ≤ 40 mm were benign.

Choy et al. [3] highlighted in their study the differences in diagnostic efficacy between biopsies of solid renal tumors and cystic lesions, as well as distinctions between FNAB and CNB. The overall diagnostic accuracy of RMB was reported—85.9% for solid tumors and 92% for cystic lesions with a solid component. Cystic lesions demonstrate lower diagnostic yield compared to solid tumors, with up to 39.8% of biopsies of these lesions being non-diagnostic. The rates of false-negative and false-positive RMB results remained low, at 3.1% and 4%, respectively. Both the sensitivity and specificity of RMB were very high. According to most of the reported studies, the difference in sensitivity and specificity between core needle biopsy and FNA shows the superiority of CNB, with an average sensitivity of 99% versus 80%. Yang et al. [23] indicated that combining both methods simultaneously enhances both diagnostic accuracy and diagnostic validity. These two techniques complement each other, each with distinct advantages and limitations. One systematic review reported a combined positive predictive value (PPV) of RMB as high as 99.8%, whereas the negative predictive value (NPV) was 68.5%.

In summary, the RMB in SRMs is an accurate method, as proved by various research groups. High sensitivity and specificity make this method valuable and may help avoid unnecessary treatment, especially surgical operations (Table 2).

## 5. Rapid On-Site Evaluation (ROSE)

To optimize the quality of the obtained material, the American Society of Cytopathology (ASC) recommends the use of rapid on-site evaluation. This procedure involves an immediate analysis of smears collected during the biopsy. ROSE enables real-time assessment of specimen adequacy and assists in guiding further diagnostic management. It allows for timely evaluation and reduces the need for repeat biopsies, thereby improving the overall quality of the sampled material. However, due to logistical and staffing challenges, this procedure is not mandatory [3,4,16].

There is limited research regarding the concordance of ROSE results with subsequent permanent specimen analysis. The diagnostic accuracy of ROSE in FNA ranges between 85% and 95%. Scanga et al. [24] reported that for CNB, ROSE sensitivity was 80.1%, specifically 72.4%, PPV 94%, and NPV 41.2%. In cystic renal lesions, 58.3% of samples were nondiagnostic on ROSE, whereas permanent sections were sufficient for diagnosis. A study comparing the concordance of ROSE assessment with the final diagnosis in CNB demonstrated agreement in 82% of cases.

Diagnostic challenges encountered during ROSE include scant or absent lesion material on touch preparations but sufficient lesional cells on core biopsies and the over-interpretation of normal elements of renal parenchyma as lesional cells at ROSE–a pitfall that has been described in proficiency testing programs. Additional possible challenges involve hepatocytes, adrenal cortical cells, histiocytes, and adipocytes. Common cells that can be mistaken for lesional cells are discussed in more detail below [3,25].

## 6. Current Guidelines Recommendations

In recent years, the rising number of incidentally detected renal masses (due to population aging and an increased use of diagnostic imaging) and the awareness of overtreatment risks have significantly increased interest in RMB as both a diagnostic and decision-making tool. Current guidelines issued by leading societies—the American Urological Association (AUA), European Association of Urology (EAU), and National Comprehensive Cancer Network (NCCN)—emphasize the growing and well-defined role of RMB in selected clinical scenarios [3,5].

The American Urological Association (AUA) recommends considering RMB in cases where the results may influence treatment decisions—particularly in patients with advanced chronic kidney disease (CKD), in candidates for active surveillance (AS), or in situations where a choice between radical and partial nephrectomy is required. RMB is strongly recommended before ablative therapies, as the tumor histology can affect ablation success and the post-treatment surveillance plan [3,26].

The EAU and NCCN issue similar recommendations, stating that RMB should be considered if there is uncertainty regarding the nature of the lesion or if the treatment strategy may be significantly influenced by histopathological results [27,28].

The importance of RMB is further supported by nationwide studies in the Netherlands, which revealed that 12% of patients who underwent nephrectomy for suspected renal cancer were ultimately diagnosed with benign tumors. This finding highlights the potential risk of overdiagnosis and unnecessary surgery. The study also indicated that older patients and those with smaller tumors (of an average of 2.9 cm) had a significantly higher likelihood of benign pathology [29].

The systematic review of PubMed, Scopus, Cochrane CENTRAL, and Web of Science databases confirmed that pre-treatment biopsy substantially reduced the number of unnecessary nephrectomies. Importantly, patients were more frequently directed towards active surveillance rather than surgical treatment. Most notably, the review found that cancer-specific survival was nearly identical between patients managed with active surveillance and those who underwent surgery. RMB demonstrates high sensitivity and specificity, comparable to or exceeding that of biopsies performed on other organs [30]. Furthermore, renal tumor biopsy (RTB) has proven to be highly accurate and can significantly guide therapeutic decisions while reducing unnecessary surgeries, with up to 27% of cases found to be benign. RTB also facilitated the detection of metastatic cancers and lymphomas, which altered management strategies [21]. RMB can also prevent inappropriate treatment in patients with metastatic malignancies. In one study, all 22 patients diagnosed with metastatic disease via RMB were spared surgery, underscoring its role in avoiding unnecessary interventions [3].

## 7. Imaging Modalities

The primary imaging modalities for evaluating renal masses are contrast-enhanced computed tomography (CT) and magnetic resonance imaging (MRI). Both allow for detailed assessment of tumor location, size, local invasion, and the presence of cystic components.

Recent developments in the field of imaging modalities include contrast-enhanced US (CEUS) as a growing alternative to CT or MRI and methods of molecular imaging, including FDG PET, PSMA PET/CT, ^99m^Tc-Sestamibi, and anti-carbonic anhydrase IX monoclonal antibodies/peptides [31]. CEUS can be primarily utilized in the kidney to evaluate complex cystic lesions, indeterminate lesions, pseudotumors (vs. solid renal tumors), renal infections, renal ischemic disorders, and renal transplants [32].

Contrast-enhanced ultrasound (CEUS) represents an emerging modality in the evaluation of renal cell carcinoma. In contrast to magnetic resonance imaging or computed tomography, CEUS enables real-time visualization, is free of ionizing radiation, and remains a cost-efficient technique, with the added benefit of being applicable at the bedside. Moreover, due to the absence of nephrotoxic effects, CEUS offers several important advantages over contrast-enhanced CT and MRI [33].

In research carried out by Tufano et al. [34], 110 patients, for a total of 118 renal masses previously identified by CT and MRI, underwent CEUS. The authors combined parameters with the highest accuracy in US B-mode (echogenicity) and in CEUS (peak intensity (PI) and the area under the curve (AUC)). This combined score yielded a PPV (positive predictive value) of 100% and an NPV (negative predictive value) of 56% with an accuracy of 81%. Finally, after removing echogenicity, the combination of PI and AUC yielded a PPV of 100% and NPV of 83%, with an accuracy of 93%. Tufano et al. concluded that CEUS parameters showed high diagnostic accuracy.

The Bosniak classification is used for stratifying malignancy risk in cystic renal lesions. However, distinguishing between benign and malignant solid tumors remains challenging, which emphasizes the need for histopathological confirmation via RMB [3,5].

Advanced imaging techniques can help differentiate certain RCC subtypes, such as clear cell versus papillary RCC, but they are not sensitive or specific enough to replace RMB [7]. Abdominal CT scans have limited specificity (70–80%) and low sensitivity (20%) for tumor detection, and they do not reliably differentiate between RCC subtypes [35].

## 8. Biopsy: FNA vs. Core Biopsy

Historically, RMB was primarily performed to exclude infections and to distinguish RCC from other malignancies, such as lymphomas, metastases, or urothelial carcinoma, which require different management approaches. Initially, the role of RMB in evaluating suspected RCC was questioned due to concerns about procedural safety, diagnostic accuracy, and the assumption that biopsy results would not significantly alter treatment decisions. Today, however, RMB is widely recognized as clinically valuable—especially for avoiding unnecessary surgery in benign cases and for better risk assessment in patients considered for active surveillance [5].

RMB techniques are divided into fine-needle aspiration (FNA) and core needle biopsy. Currently, core biopsy is preferred due to its superior diagnostic performance, with a sensitivity of 99.1% and a specificity of 99.7%, compared with 93.2% and 89.8% for FNA [3,5]. While core biopsy provides more tissue for histological and immunohistochemical analysis, FNA may be a safer choice for patients with increased bleeding risk or when the lesion is located in a difficult-to-access area. Combining both techniques can improve diagnostic accuracy and reduce the rate of nondiagnostic results [3]. Marconi et al. [15] demonstrated that core biopsy yields better diagnostic outcomes than FNA, with sensitivity and specificity rates of 99.1% and 99.7%, respectively, compared with 93.2% and 89.8% for FNA. Therefore, core samples obtained with 18-gauge needles or larger are recommended instead of thinner (20-gauge or smaller) needles typical for FNA. It is also emphasized that in cases of nondiagnostic results, repeat RMB often leads to a conclusive diagnosis [5].

## 9. Future Directions

The increasing incidence of incidentally discovered renal masses, often detected through imaging for unrelated conditions, has significantly broadened the clinical application of preoperative RMB. Once reserved for select cases, RMB is now widely accepted as a diagnostic tool that can guide personalized treatment planning. This shift reflects the growing recognition that not all renal masses require surgical intervention and that accurate preoperative diagnosis can significantly influence patient outcomes [3,12]. Recent advances in renal tumor classification have been instrumental in enhancing the value of RMB. The integration of traditional morphology with ancillary diagnostic tools—such as immunohistochemistry (IHC) and molecular studies—has allowed for more precise characterization of renal tumors. These tools help pathologists understand not only the histological subtype but also the molecular underpinnings of the disease, which are increasingly linked to clinical behavior and prognosis. This integrative approach allows for more informed risk stratification and tailored treatment strategies [36]. Although morphology alone often provides sufficient information to diagnose common renal tumors (e.g., clear cell RCC, papillary RCC), additional studies are often necessary to further classify ambiguous cases or identify recently recognized variants. Ancillary testing is especially critical for tumors that exhibit overlapping cytologic features or when the morphology suggests a non-epithelial process, such as lymphoma, metastasis, or granulomatous inflammation. Immunocytochemistry or immunohistochemistry can usually be performed on cell blocks or formalin-fixed samples from RMB, while molecular studies may reveal mutations or translocations that confirm specific diagnoses [3].

The fifth edition of the WHO classification of renal tumors, published in 2022, emphasizes the importance of obtaining adequate tissue for these ancillary techniques. A significant update in this edition is the inclusion of a new category for molecularly defined renal neoplasms, such as TFE3-rearranged renal cell carcinoma and ELOC-mutated RCC. These entities are often indistinguishable by morphology alone, reinforcing the need for comprehensive molecular testing. RMB provides a minimally invasive route to obtain the tissue required for such testing, reducing the need for upfront nephrectomy in cases where diagnosis is uncertain [37,38]. Biopsy allows for differentiation of benign from malignant tumors, exclusion of metastases, hematological and inflammatory processes, and provides prognostic information such as tumor subtypes and nuclear grade [4,21]. It can also reveal genetic aberrations in RCC, aiding in better risk stratification and guiding targeted therapies [5].

While current therapeutic decisions for RCC are not yet fully driven by molecular profiling, the landscape is rapidly evolving. Advances in immunotherapy, particularly the use of checkpoint inhibitors, have opened new avenues for treating advanced or metastatic RCC. Molecular profiling is anticipated to play a growing role in predicting response to these therapies and selecting appropriate patient populations [3,6]. In this evolving context, cytopathology laboratories are emerging as central players in delivering precision medicine. Their responsibilities now extend beyond slide interpretation to include the optimization of RMB processing, triaging, and integration of results with clinical management plans. ROSE allows cytopathologists to provide immediate feedback during procedures, enhancing sample quality and facilitating timely, accurate diagnosis [3]. Cytopathologists are also instrumental in guiding the direction of the biopsy, providing feedback directly to the interventional radiologist. This collaboration facilitates the collection of high-quality samples and minimizes the number of needle passes required, reducing patient discomfort and the risk of complications. Importantly, based on the cytomorphologic findings during ROSE, cytopathologists can also make crucial decisions about triaging the sample for additional testing, such as flow cytometry in hematologic neoplasms, immunohistochemistry in metastatic tumors, or molecular studies in cases suspicious for specific genetic alterations [3,16].

The integration of ROSE into the renal mass biopsy workflow significantly enhances the diagnostic yield of the procedure. By allowing immediate evaluation of sample adequacy and cellular composition, ROSE increases the likelihood of obtaining representative material from heterogeneous tumors. It also ensures that sufficient tissue is reserved for downstream ancillary studies, which are essential for the precise classification of renal neoplasms, especially newly defined molecular subtypes. This proactive approach not only reduces the frequency of non-diagnostic or insufficient samples but also minimizes the need for repeat biopsy procedures. Furthermore, ROSE enables timely clinical decision-making by expediting the diagnostic process and facilitating early therapeutic planning. In complex cases, such as those involving suspected infectious, inflammatory, or hematologic conditions, real-time guidance is invaluable in ensuring appropriate and targeted testing is performed without delay, ultimately improving diagnostic accuracy, treatment efficiency, and patient outcomes [16].

## 10. Conclusions

In conclusion, renal mass biopsy (RMB) has become an increasingly important tool in the evaluation and management of incidentally detected renal masses. Supported by leading international guidelines, RMB offers high diagnostic accuracy, reduces unnecessary nephrectomies, and facilitates tailored treatment strategies by distinguishing benign from malignant lesions and identifying metastatic disease. It is a safe procedure with a low complication rate, yet it remains underutilized due to entrenched surgical habits and the lack of fully standardized protocols. Broader implementation of RMB and consistent adherence to guidelines could significantly improve patient care by minimizing overtreatment, avoiding unnecessary surgeries, and ensuring more precise therapeutic decision-making.

## Figures and Tables

**Table 1:** Renal tumor biopsy complications.

Author and Year	Number of Patients	Seeding of the Tumor	Complication Types No. (%)			Overall Complication Rate (%)
			Grade A	Grade B	Grade D	
**Mazin Hashim et al. (2024)** [18]	193	0	1 (0.5%)—self-limiting subcapsular hematoma	0	1 (0.5%)—haemothorax	1%
**Serhal et al. (2024)** [16]	167	NR	6 (3%)—perinephric hematoma, 1(0.5%)—retroperitoneal hematoma	1 (0.5%)—pain	1 (0.5%)—pyelonephritis	5%
**Isgrò et al. (2023)** [12]	225	1 (0.4%)	NR	NR	NR	4.8%
**Gao et al. (2023)** [4]	168	0	3 (1.8%)—hematuria	3 (1.8%)—severe pain 1 (0.6%)—infection	5 (3.0%)—hemorrhage	7.2%
**Okhunov** **et al.** **(2021)** [19]	72	0	0	0	0	0%
**Bada et al.** **(2021)** [14]	94	0	3 (3%)—prevalently local hematoma treated with surveillance	0	0	3%
**Tong et al.** **(2020)** [17]	83	0	13 (15.6%)—hematuria was the most common complication	2 (2.4%)	0	18.1%
**Patel et al.** **(2016)** [20]	2979	NR	119 (4.2%)—perirenal hematoma	36 (1.2%)—flank pain	18 (0.6%)—pneumothorax, 12 (0.4%)—hemorrhage	8%—median (IQR: 2.7–11.1%)
**Marconi et al. (2016)** [15]	5228	1 (0.0019%)	NR	37 (0.7%) bleeding requiring pharmacologic treatment, intervention, or blood transfusion		NR
**Richard et al. (2015)** [13]	509	0	24 (4.7%)—perinephric hematoma	NR	NR	8.5%

NR—Not reported on studies, Grade A—minor complications not requiring intervention, Grade B—complications requiring pharmacological treatment, Grade D—infectious complications.

**Table 2:** Overview of cited RMB studies.

Author and Year	Time Period	Mean Tumor Size (cm)	Number of Patients	Sensitivity	Specificity	Positive Predictive Value	Negative Predictive Value
**Mazin Hashim et al. (2024)** [18]	2000–2017	≤4 cm (SRM)	193	95%	100%	100%	92%
**Azawi et al. (2018)** [21]	2014–2017	≤ 4 cm (SRM)	61	98%	100%	NR	NR
**Patel et al.** **(2016)** [20]	Systematic review of 20 studies, 1997–2015	≤4 cm (SRM)	2979	97.5%	96.2%	99.6%	63.3%
**Marconi et al. (2016)** [15]	Systematic review of 57 studies, 1946–2014	2.4–9.2 cm	5228	99.1% (CNB) 93.2% (FNAB)	99.7% (CNB) 89.8% (FNAB)	NR	NR

NR—Not reported on studies, CNB—core needle biopsy, FNAB—fine needle aspiration, SRM—small renal masses—contrast-enhancing solid masses detected on diagnostic imaging, with a size of less than or equal to 4 cm.

## Abbreviations

AS	Active surveillance
ASC	American Society of Cytopathology
AUA	American Urological Association
CCRCC	Clear cell renal cell carcinoma
CNB	Core needle biopsy
CKD	Chronic kidney disease
CT	Computed tomography
EAU	European Association of Urology
FNA	Fine-needle aspiration
FNAB	Fine-needle aspiration biopsy
MRI	Magnetic resonance imaging
NCCN	National Comprehensive Cancer Network
RCC	Renal cell carcinoma
ROSE	Rapid on-site evaluation
RMB	Renal mass biopsy
RTB	Renal tumor biopsy
SRM	Small renal masses—contrast-enhancing solid masses detected on diagnostic imaging, with a size of less than or equal to 4 cm
IHC	Immunohistochemistry
